# Case Report: Unravelling the Mysterious Lichtenberg Figure Skin Response in a Patient With a High-Voltage Electrical Injury

**DOI:** 10.3389/fmed.2021.663807

**Published:** 2021-06-11

**Authors:** Andrew Lindford, Susanna Juteau, Viljar Jaks, Mariliis Klaas, Heli Lagus, Jyrki Vuola, Esko Kankuri

**Affiliations:** ^1^Department of Plastic Surgery, Helsinki Burn Centre, Helsinki University Hospital, University of Helsinki, Helsinki, Finland; ^2^Department of Pathology, Haartman Institute, University of Helsinki and Helsinki University Hospital Diagnostic Center, HUSLAB, Helsinki, Finland; ^3^Institute of Molecular and Cell Biology, University of Tartu, Tartu, Estonia; ^4^Helsinki Wound Healing Centre, Helsinki University Hospital, Helsinki, Finland; ^5^Department of Pharmacology, Faculty of Medicine, University of Helsinki, Helsinki, Finland

**Keywords:** electrical injury, lactoferrin, Lichtenberg figures, skin, tissue response

## Abstract

We describe a case of Lichtenberg Figures (LFs) following an electrical injury from a high-voltage switchgear in a 47 year-old electrician. LFs, also known as ferning pattern or keraunographic markings, are a pathognomonic skin sign for lightning strike injuries. Their true pathophysiology has remained a mystery and only once before described following an electical injury. The aim was to characterise the tissue response of LFs by performing untargeted non-labelled proteomics and immunohistochemistry on paraffin-embedded sections of skin biopsies taken from the area of LFs at presentation and at 3 months follow-up. Our results demonstrated an increase in dermal T-cells and greatly increased expression of the iron-binding glycoprotein lactoferrin by keratinocytes and lymphocytes. These changes in the LF-affected skin were associated with extravasation of red blood cells from dermal vessels. Our results provide an initial molecular and cellular insight into the tissue response associated with LFs.

## Introduction

Lichtenberg figures (LFs), also known as ferning pattern, feathering, keraunographic markings or arborescent burns, are a pathognomonic skin sign for a lightning strike injury (1). They received their name from Georg Lichtenberg, who in 1777 first described them whilst conducting static electricity experiments. The true pathophysiology of LFs remains unknown (2, 3). LFs appear to be a mysterious infrequent, transient sequelae of lightning strike injuries and have been sporadically reported in several case reports (1–10). They arise from a positive discharge on the skin that, in the case of negatively charged lightning, is speculated to be generated by flashover from a nearby protrusion at earth potential (11). Our group recently reported a case of LFs following a lightning strike injury and discussed its possible pathophysiology (10, 12).

Here, we describe the protein-level molecular characterisation of LFs in a patient that suffered a high-voltage electrical injury from an alternating current switchgear and presented with non-blanching red marks consistent with LFs over his right flank. We performed untargeted non-labelled proteomics and immunohistochemistry on paraffin-embedded biopsy sections of LF-affected skin in comparison to follow-up skin samples to unravel the molecular and histological pathobiology of the tissue response at the time of LFs.

## Case Presentation

**Figure 1:**
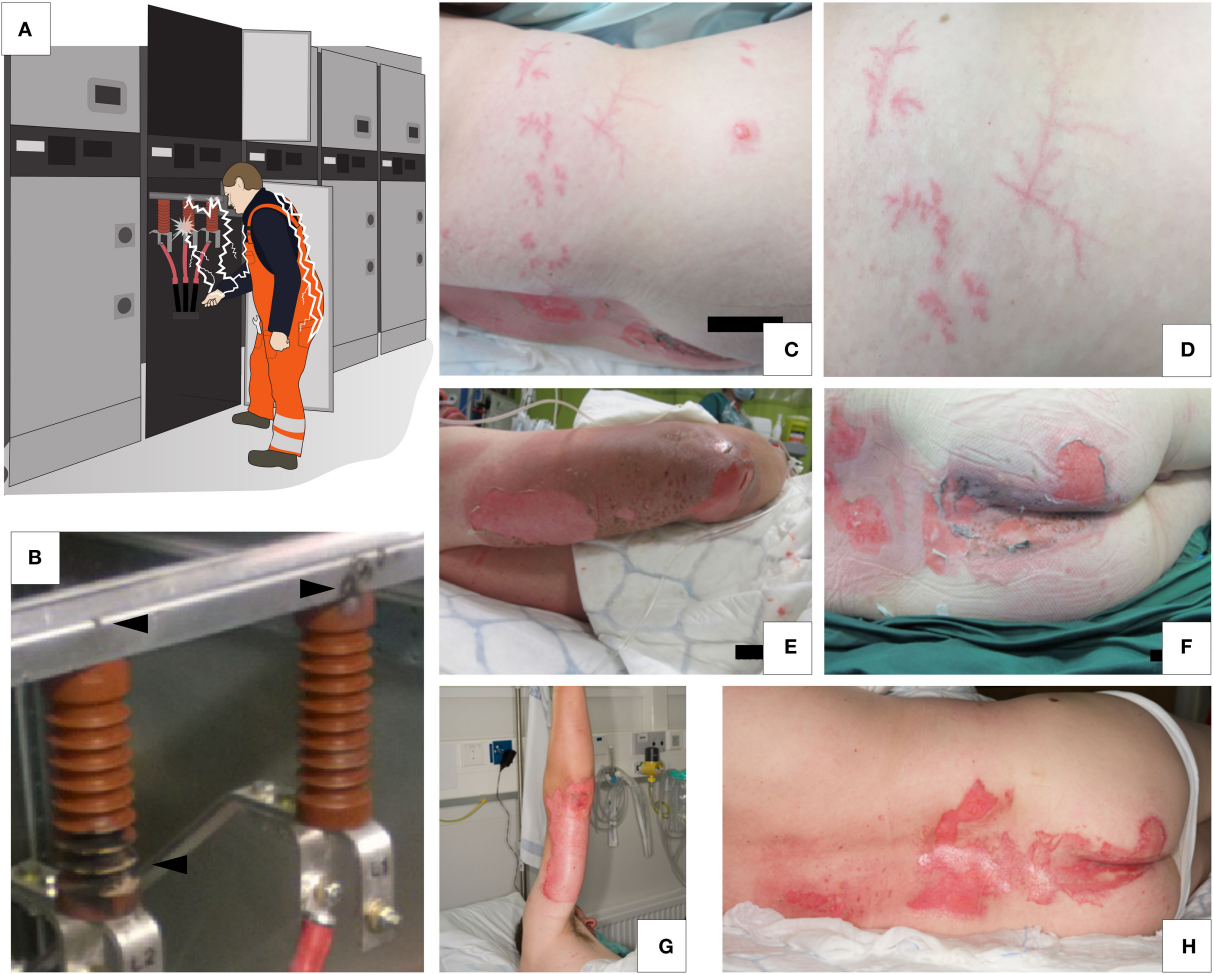
**(A)** Mechanism of the electric arc injury from the 20 kV medium-voltage switchgear at inspection. **(B)** Light-arc and electric discharge-induced damage marks in the switchgear. **(C,D)** Lichtenberg figures on the right flank. **(E,F)** Second-degree dermal burns involving right arm, lower back and buttocks. **(G,H)** Burn wounds on right arm, lower back and buttocks after 3 days. Lichtenberg figures not present.

A 47-year-old otherwise healthy electrician sustained a high voltage electrical burn whilst at work commissioned to extend a 20 kV outgoing bay unit in an air-insulated high-voltage switchgear. He was examining the connexions within the switchgear and incorrectly assumed the “bay unit” to be de-energised. He directed his hand towards one of the 20 kV outgoing units and was “struck” by electricity from electrical arcing (Figure 1A). Of note, he had a large metal combination wrench (for 19 mm nuts) in his right front hip pocket that may have influenced the injury pattern. There were signs of damage inside the bay unit with the earthing circuit connector indicating that earthing of electric current occurred at least partially, over a very short distance, when the patient's hand or the combination wrench initiated the electrical arc (Figures 1A,B). The patient was able to walk out from the switchgear.

Upon arrival of the paramedics, he was alert and orientated and did not report a fall or loss of consciousness. He was able to walk to the ambulance unassisted and was taken to the nearest emergency department. He had a second-degree dermal burn covering 7% of total body surface area involving his right elbow, arm, lower back, and buttocks (Figures 1C–F).

He was then transferred to the burn centre where upon further examination he was found to have a normal neurological status in all limbs and no clinical signs of compartment syndrome or deeper muscle injury. In addition, several non-blanching red marks consistent with LFs were observed over his right flank (Figures 1C–F). Blood tests revealed normal concentrations of haemoglobin (140 g/L), C-reactive protein (<3 mg/L) and creatinine (75 μmol/L). Blood cell counts for leukocytes (11.5 × 10^9^ cells/L) and neutrophils (7.41 × 10^9^ cells/L) were elevated, consistent with an acute inflammatory reaction. In addition, there were only very slight increases in myoglobin and creatinine concentrations (194 ng/mL and 316 U/L, respectively), ruling out a severe muscle injury.

Within the following 24 h both the white cell count and the myoglobin concentration normalised (6.9 × 10^9^ cells/L and 49 ng/mL, respectively), as did the neutrophil count and creatine kinase levels. He was monitored for 2 days on the high dependency unit and was administered simple analgesia (ibuprofen 600 mg tds and paracetamol 1g tds). The burns were dressed with an antimicrobial exudate transfer layer (Mepilex® Transfer Ag, Mölnlycke Health Care AB, Gothenburg, Sweden) and were redressed every 3–5 days. The LFs disappeared completely within 24 h (Figures 1G,H showing status after 3 days) and the burns healed within 3 weeks. He returned to work 6 weeks after injury.

## Skin Biopsies and Analysis

After obtaining a full written informed patient consent, a 4-mm punch biopsy was taken at the time of admission to the Helsinki Burn Centre from the erythematous area of LFs over the right flank. A follow-up biopsy sample was taken 3 months after the injury from the healed area of skin adjacent to the still visible minor scar from the earlier biopsy site. Both tissue samples were analysed histopathologically and underwent proteomics analysis and immunohistochemical stainings. Protein expression was ultimately compared between samples obtained from the site of injury containing the LFs and at the 3-month follow-up.

Detailed additional methodology is provided in the Supplementary Methods.

## Results

**Figure 2:**
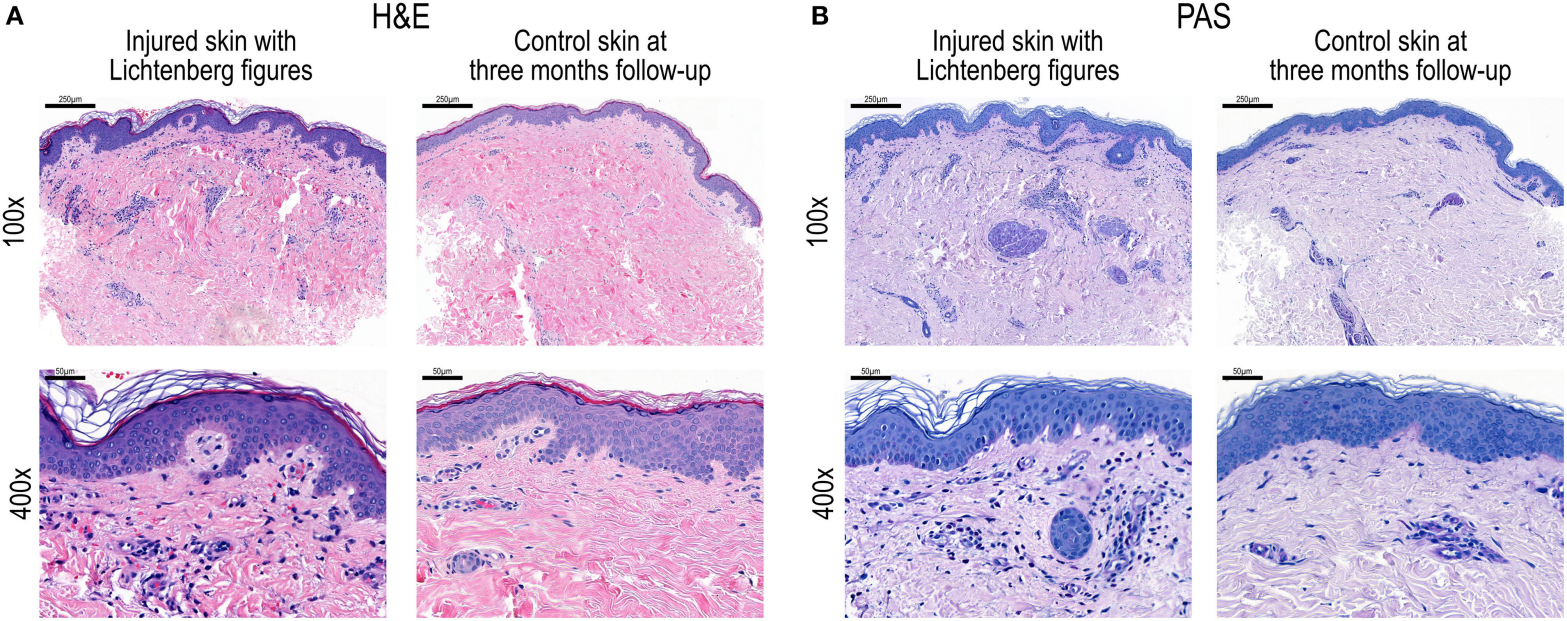
**(A)** Haematoxylin and Eosin (H&E) staining and **(B)** Periodic acid-Schiff (PAS) staining of skin biopsy sections after electric injury and Lichtenberg figures and at 3 months follow-up as control. Magnifications 100 × and 400×.

Histopathological comparison of injury and control samples (Figures 2A,B) revealed prominent lymphocytic infiltrates and red blood cell extravasation around the small blood vessels in the upper dermis of the injury sample. Despite evident tissue infiltration by lymphocytes, Periodic acid-Schiff (PAS) staining showed intact vessel walls and absent vasculitis (Figure 2B).

**Figure 3:**
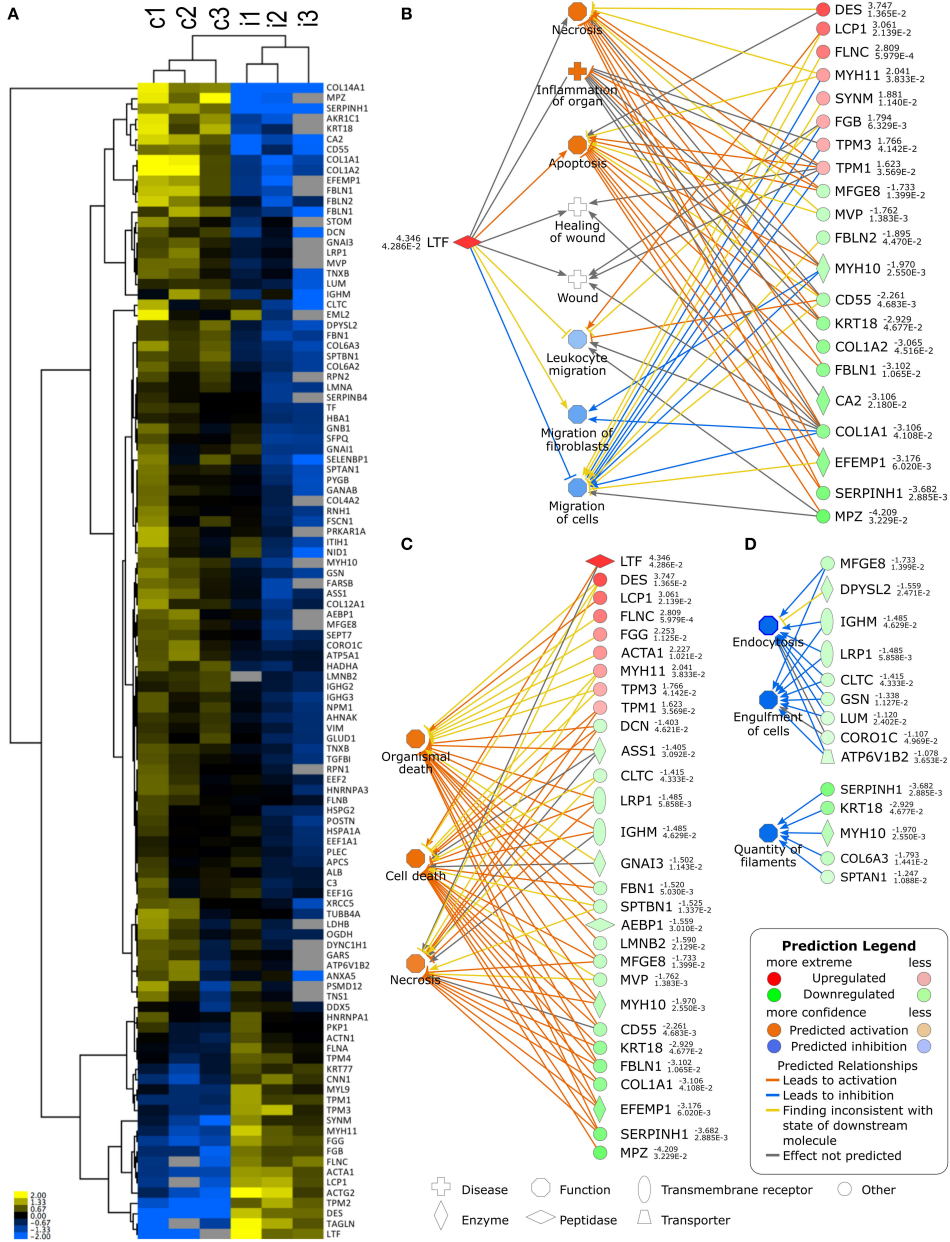
**(A)** Heatmap clustering of differentially expressed proteins in three consecutive tissue sections of 3 month follow-up control (c1-3) and injured (i1-3) biopsy samples. **(B)** Lactoferrin (LTF)-associated activated and inhibited pathways and identified differentially expressed proteins (DEPs) associated to each. **(C)** Top 3 predicted upregulated pathways and their associated DEPs. **(D)** Top 3 predicted inhibited pathways and their associated DEPs. Differentially expressed proteins and associated signalling pathways were analysed using Ingenuity Pathway Analysis (IPA).

Sequential sections were then cut from the paraffin-embedded biopsy samples for untargeted proteomic characterisation of the molecular tissue response at the site of the LFs. To obtain the most reliable view possible, three sequential sections from both injured and follow-up control were analysed (Figure 3). We identified differentially expressed proteins (DEPs), such as lactoferrin (lactotransferrin, LTF), at a higher abundance in the injured tissue sample in contrast to the control sample taken at 3-month follow-up. Full lists of the DEPs identified are provided in the Supplementary Tables 1, 2. Figure 3 shows an expression heatmap of DEPs between the three consecutive biopsy section sets from injured skin with LFs and follow-up control. Comparative analysis of the injured tissue sample with the control follow-up sample using Ingenuity Pathway Analysis (IPA) software identified LTF-associated functions “Necrosis, Inflammation of organ and Apoptosis” to be significantly activated in the injured tissue sample, whereas cell and leukocyte migration were predicted to be suppressed (Figure 3B). Figure 3C shows the DEP associations to the three most significantly activated or suppressed pathways as predicted by IPA. The analysis shows activation of IPA-pathways for Organismal death, Cell death and Necrosis and inhibition of pathways for Endocytosis, Engulfment of cells and Quantity of filaments. Taken together, the DEPs identified by proteomics and their subsequent pathway analysis suggests increased activity of pathways for cell death and immunosuppressive activity in the electric-arc-injured skin. According to this analysis, LTF is the topmost candidate for further analysis. Complete results from the pathway analysis are listed in Supplementary Table 3.

**Figure 4:**
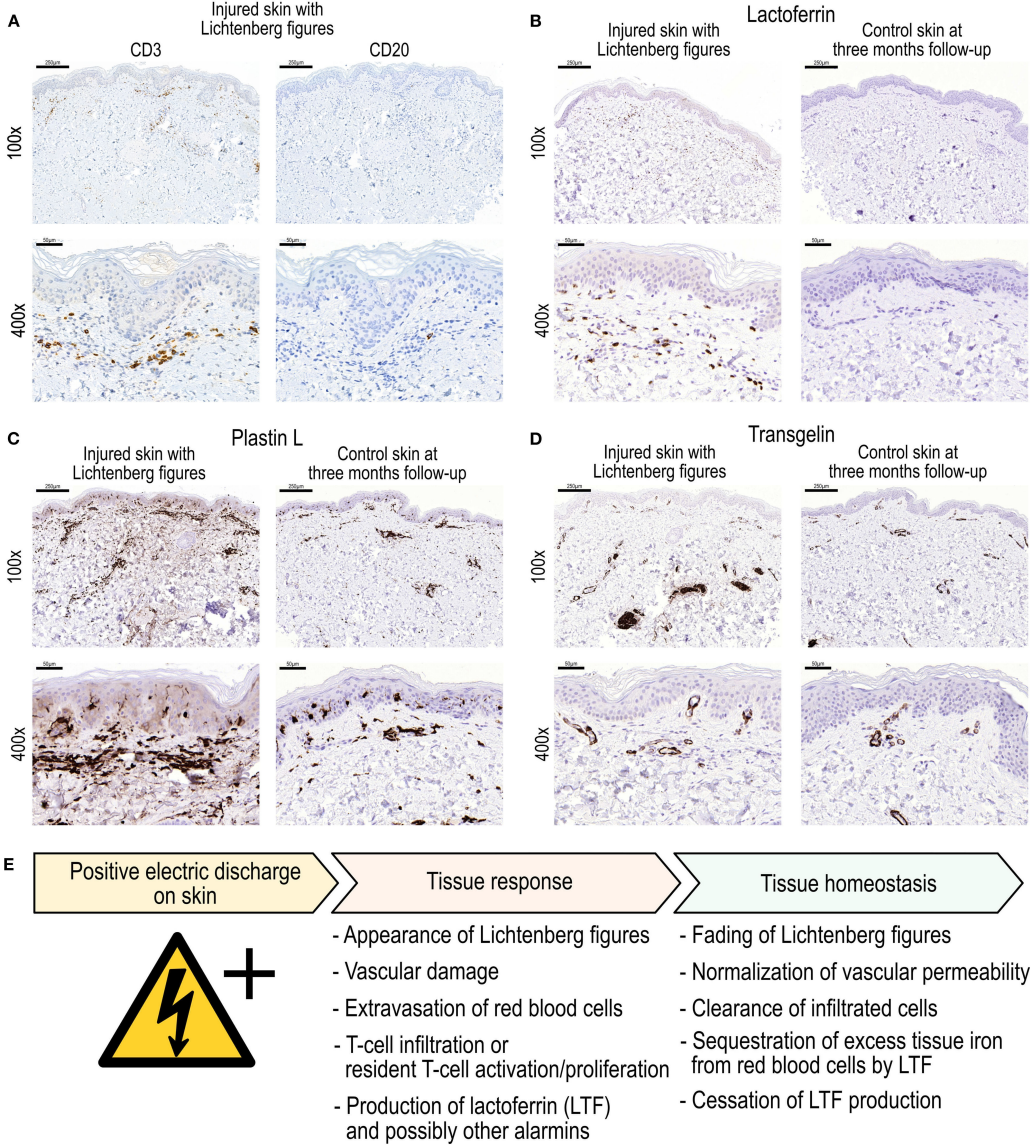
**(A)** Immunohistochemical (IHC) staining for CD3 (general T-cell marker) and CD20 (general B-cell marker) in biopsy section from electric-arc-injured skin. **(B–D)** demonstrate IHC staining for selected differentially expressed proteins as discovered in the proteomics analyses. Specifically, IHC stainings for (**B)** Lactoferrin, **(C)** Plastin L, and **(D)** Transgelin in skin biopsy sections after high voltage injury and at 3-month follow-up control. Magnifications 100× and 400×. **(E)** Proposed sequence of events after electric discharge on the skin associated with the appearance and fading of Lichtenberg figures.

The lymphocytic infiltrate consisted mainly of mature T-lymphocytes (CD3^+^) and a few solitary CD20^+^ B-cells (Figure 4A). Some LTF staining was also present in keratinocytes and endothelial cells in the sample from injured skin with LFs. Increased staining for L-plastin, a key protein in T-cell signalling, motility and trafficking was found in the dermis of the injured skin sample as compared to the follow-up control skin sample. However, tissue staining for another DEP identified by proteomics, transgelin, an actin-binding protein that has been implicated in the regulation of T-cell activation (13), showed no marked differences between samples.

## Discussion

LFs associated with a high-voltage injury has only once been previously described (14). Furthermore, we present here, to our knowledge, the first instance in which a biopsy of a LF skin lesion was taken from a patient who survived, thus enabling a control biopsy 3 months later. Using these paired samples from a single patient, we herein provide for the first time molecular insight at the protein level to the tissue response associated with LFs. Our findings encourages the use of molecular techniques on samples collected even from rare cases. A prospective multicentre study design, an active lookout for patients presenting with LFs, dedicated sampling protocols for specific untargeted downstream molecular and histological analyses are needed to enable further characterisation of LFs tissue response in detail.

Interestingly, Resnik and Wetli (5) reported a normal epidermis, normal dermis and only focal extravasation of blood in the upper subcutaneous tissue in a biopsy sample from a deceased victim of lightning strike who exhibited LFs. Our study revealed a significantly greater level of expression of the glycoprotein LTF and CD3^+^ leukocyte extravasation in the erythematous marks of LFs than in the control sample obtained at follow-up 3 months later (Supplementary Table 3, Figure 4). LTF is a secreted transferrin homologue protein produced by immune and most epithelial cells (15). It binds and sequesters iron even under inflammatory in tissue injury conditions with low pH (16). In addition to its iron binding characteristics, it is involved in numerous biological processes including immunomodulation, leukocyte chemotaxis and also possesses antimicrobial properties (17–20). After tissue injury, LTF acts as a molecular alarmin in innate and adaptive immunity contributing to leukocyte recruitment and dendritic cell maturation (21). LTF may also stimulate *in vivo* angiogenesis via the upregulation of KDR/Flk-1 expression in endothelial cells (22). LTF contributes to T-helper cell polarisation and has a profound modulatory action on the adaptive immune system by promoting the maturation of T-cell precursors into competent helper cells. The adaptive immune response is dominated by T-cell (CD3^+^) activity (23). Furthermore, LTF can influence cutaneous immune and inflammatory processes secondary to regulation of the production of cytokines (20). LTF has been attributed with a homeostatic role to limit pathological development by, for example, sequestering iron and reactive oxygen intermediates (24). Moreover, LTF has been shown to promote wound healing through promotion of fibroblast activity and keratinocyte proliferation and migration (25). Hence we may surmise that the electric injury induced vascular leakage and extravasation of haeme-iron-loaded red blood cells; this contributed to the tissue's response to produce LTF for helping to deal with the tissue iron overload. LTF, produced by the immune cells and keratinocytes, then subsequently limits tissue injury as well as bacterial growth (15).

As a first response in innate immunity, one would expect to find neutrophils and macrophages in the tissue. However, we found a CD3^+^ T-cell infiltrate accompanied by an occasional CD20^+^ B-cell in the LF-affected skin sample. The skin's unique innate immunity and tolerance resting predominantly on resident T-cells rather than recruitment of circulating immune cells (26). For the generation of LFs, a positively charged discharge in the skin, in this case electric arcing from high voltage switchgear, damages the skin integrity of dermal small vessels and leads to extravasation of red blood cells. The electric injury most likely also influences resident skin cells, especially keratinocytes and lymphocytes. The electric damage to the low-resistance fluid-filled blood vessels manifests as disruption of vascular wall integrity followed by red blood cell leakage into the tissue. The tissue response is associated with the production of LTF, to both signal the alarm for tissue injury and, on the other hand, to induce repair and limit further damage after electrical injury. Based on our findings, we propose an event sequence as shown in Figure 4E after electric discharge on the skin surface leading to the formation of Lichtenberg figures.

Amongst other upregulated proteins of note (Figure 3; Supplementary Table 1) the bacterial permeability increasing protein and fibrinogen components (beta and gamma chains) can also participate in the innate defence reaction at an injured site (27, 28). Unsurprisingly, several components of the cytoskeleton (actin alpha and gamma isoforms, myosin-11, desmin and tropomysin alpha-1, -3, and -4 and beta chains) as hallmarks of physical cell damage, were also expressed more abundantly at the acutely injured site.

In spite of the potential effect of high temperature, the 12kDa member of the heat-shock 70 protein family, HSPA12A, was less abundant in the LF-affected than in the follow-up control skin. Also several collagen isoforms (alpha-1 chains from collagens I, III, and XIV and alpha-2 chains from collagen I) were found at lower levels in the acutely injured skin. It is possible that this could reflect electricity-induced damage to the extracellular matrix or a compensative recovery response to burn injury.

The LFs have attracted numerous theories on their transient nature as well as pathogenesis (1–5, 11). Ten Duis et al. (11) demonstrated that the non-anatomical ferning pattern of LFs is a first degree burn caused by a positive discharge in the skin (12). Interestingly, lightning and electric burns may be caused by various forms of radiation including thermal radiation as well as ionising radiation that as a positive discharge-even in ambient air-develops a characteristic ferning pattern (29). With negative discharge, Ten Duis et al. observed a less random and more deterministic or homogenous pattern (11). Interestingly, our patient suffered from electric discharge arising from switchgear with alternating currents. From the photographs (Figures 1C,D) one can speculate the patterns on the left to have arisen from negative discharge while the fractal, ferning pattern on the right (lower flank) demonstrates a typical LF. It can be speculated that the patient's skin LFs arose with a positive discharge from the switchgear either during an appropriate voltage polarity or that the LFs on the right flank derive from a positive flashover to which e.g., the wrench that the patient carried in his right hip pocket could have contributed.

By utilising untargeted proteomics on clinical samples taken at presentation and after healing, we provide further insight into LFs as a transient physical and pathological phenomenon. We associate a specific molecular tissue response, vascular injury, red blood cell extravasation, and CD3^+^ T-cell accumulation with the positive electric discharge that generated LFs on the skin. Of particular interest is the expression of LTF and the T-cell abundance at the site of injury. It is tempting to speculate that the mitogen-dependent stimulation of T-cell proliferation by iron (30), presumably presented in the LF-affected tissue in the form of extravasated red blood cell-contained haeme iron, may contribute to local T-cell responsivity (31). In turn, LTF would then act as a homeostatic control of the T-cell response.

In conclusion, we postulate that an innate alarm response from the positive electric discharge-induced skin burn is triggered by resident keratinocytes and immune surveillance T-cells (Figure 4E). The dermal vasculature is damaged by the discharge leading to extravasation of red blood cells that may contribute to the appearance of LFs. The tissue LTF response serves multiple purposes ranging from fine tuning and locally limiting the innate immune response to sequestration of extravasated red-blood-cell-derived iron. Our results suggest a central role for T-cells and a LTF-mediated tissue response after electric discharge on skin with LFs.

## Data Availability Statement

Data are available via ProteomeXchange with identifier PXD011532.

## Ethics Statement

Written informed consent was obtained from the patient for the publication of any potentially identifiable images or data included in this article.

## Author Contributions

AL, JV, and EK contributed to conception and design of the study. EK organized the database. AL and EK wrote the first draft of the manuscript. MK and VJ performed the histological section proteomics analysis. All authors contributed to the analysis of the data, contributed to manuscript revision, read, and approved the submitted version.

## Conflict of Interest

The authors declare that the research was conducted in the absence of any commercial or financial relationships that could be construed as a potential conflict of interest.
