# Supplementary material for: Case Report: Unravelling the Mysterious Lichtenberg Figure Skin Response in a Patient With a High-Voltage Electrical Injury
Source: Front Med (Lausanne). 2021 Jun 11;8:663807. doi: 10.3389/fmed.2021.663807 (PMC8226253; doi:10.3389/fmed.2021.663807)
Supplement: Supplementary file 1 [file Data_Sheet_1.docx]

**Supplementary methods**

*Sample section lysis—*Three formalin-fixed paraffin-embedded 10-µm-thick sections from each sample block were deparaffinized by incubation at room temperature in xylene for 10 min. The deparaffinized tissue sections were then rehydrated with a graded series of ethanol and briefly air-dried. The tissue sections were lysed in a buffer consisting of 0.3 M Tris-HCl, pH 8.0, 0.1 M DTT, 0.5% (w/v) polyethylene glycol 20 000, and 4% SDS. The samples were incubated at 99 °C for 90 min as described previously [1,2].

*Mass-spectrometry—*Samples were purified by methanol/chloroform protein precipitation. Protein reduction and alkylation was performed in 100 mM Tris (pH 8.0) containing 5% sodium deoxycholate by adding 5 mM DTT and 10 mM iodoacetamide for 20 min at room temperature. Protein digestion was performed by diluting the solution 5x with mQ H_2_O, prior to adding 400 ng trypsin (Pierce, Thermo Fisher Scientific, Waltham, MA, USA) per reaction and by incubating overnight at +37°C. The digestion was then stopped by adding trifluoroacetic acid to a concentration of 0.5%. The peptides were purified by C18 StageTips (3M Empore, Eagan, MN, USA). Peptides were analyzed on an Ultimate 3000 RSLCnano (Dionex, ThermoFisher Scientific) nano-LC system with a C18 cartridge column (Dionex) and an in-house packed (3 µm ReproSil-Pur C18AQ particles, Dr. Maisch HPLC GmbH, Ammerbuch, Germany) 50 cm 75 µm ID emitter-columns (New Objective, Woburn, MA, USA) using a 60 min 8-50% B gradient where buffer A was 0.1% formic acid in water and B, 0.1% formic acid in 80% acetonitrile. Separated peptides were eluted at 200 nl/min (spray voltage 2.4 kV) to a Q Exactive Plus (ThermoFisher Scientific) mass-spectrometer operating with a top-5 MS/MS strategy with a 0.6 s cycle time. Dynamic exclusion was set to 30 s. Only charge states +2 to +6 were analyzed. Raw data were identified and quantified with MaxQuant 1.4.0.8 software package. Search was performed against UniProt (www.uniprot.org) human database using the tryptic digestion rule. Proteomics raw data from each sample normalized to its detected proteins’ average intensity. These sample tissue area- and intensity-normalized values were then used in subsequent analyses using a statistically significant cutoff of p<0.05 for each identified protein in any comparison between groups. Clustering of proteins and sample groups was performed using Gene Cluster 3.0 software [3], and heat map visualizations were produced using Java TreeView 1.16r4 software [4].

*Pathway analysis*—Pathway analysis was carried out as previously described [2]. Briefly, pathway analysis was conducted using the Ingenuity Pathway Analysis software (IPA, Qiagen Bioinformatics, Redwood City, CA8). IPA and Ingenuity’s Knowledge Base were utilised to algorithmically generate functional connectivity networks to identify both biological functions and diseases with greatest significance to the data set.

*Immunohistochemistry*—Histological sectioning, hematoxylin&eosin (H&E) and periodic acid–Schiff (PAS) stainings as well as immunohistochemistry for CD3 and CD20 were carried out using corresponding standard clinical staining protocols at an EN ISO 17025 and 15189-accredited laboratory (HUSLAB, Helsinki, Finland). Additional immunohistochemistry was performed at BioSiteHisto Ltd (Tampere, Finland). Samples were analysed for targeted protein immunoreactivity using anti-lactoferrin (1:2000, HPA059976, Sigma-Aldrich, Merck KGaA, Darmstadt, Germany), anti-plastin-L (1:1000, ab109129, Abcam plc, Cambridge, United Kingdom) or anti-transgelin (1:100, 60213-1-Ig, Clone 2A10C2, Proteintech Group Inc, Rosemont, IL, USA) primary antibodies. Scanning of microscopy slides was carried out by the Digital microscopy and molecular pathology unit at the Finnish Institute for Molecular Medicine (FIMM, Helsinki, Finland) or by BioSiteHisto Ltd. Pannoramic Viewer software (3DHISTEC Ltd, Budapest, Hungary) was used for image capture.

*Statistics*—A p<0.05 cutoff was used to assign significance and to select proteins of interest for analysis using IPA. IPA software-assisted analysis and right-tailed Fisher’s exact test was used to calculate a p-value determining the probability that each biological function and/or disease assigned to that data set is due to chance alone.

**References to Supplementary methods**

1. Kawashima Y, Kodera Y, Singh A, Matsumoto M, Matsumoto H (2014) Efficient extraction of proteins from formalin-fixed paraffin-embedded tissues requires higher concentration of tris(hydroxymethyl)aminomethane. Clin Proteomics 11:4. <https://doi.org/10.1186/1559-0275-11-4>
2. Lagus H, Klaas M, Juteau S, Elomaa O, Kere J, Vuola J, Jaks V, Kankuri E (2019) Discovery of increased epidermal DNAH10 expression after regeneration of dermis in a randomized with-in person trial - reflections on psoriatic inflammation. Sci Rep (article in press). <https://doi.org/10.1038/s41598-019-53874-z>
3. de Hoon MJ, Imoto S, Nolan J, Miyano S (2004) Open source clustering software*.* Bioinformatics 20:1453-1454. <https://doi.org/10.1093/bioinformatics/bth078>
4. Saldanha AJ (2004) Java Treeview--extensible visualization of microarray data*.* Bioinformatics 20:3246-3248. <https://doi.org/10.1093/bioinformatics/bth349>
